# Shoulder Joint Infections with Negative Culture Results: Clinical Characteristics and Treatment Outcomes

**DOI:** 10.1155/2019/3756939

**Published:** 2019-02-12

**Authors:** Mohamed Attia Abdou, Ahreum Jo, Ik-Sun Choi, Chae-Jin Iim, Hyeng-Kyu Park, Hee-Kyun Oh, Sung-Min Kim, Myung-Sun Kim

**Affiliations:** ^1^Department of Orthopaedics, Chonnam National University, College of Medicine, Gwangju 61469, Republic of Korea; ^2^Department of Biomedical Science, Chonnam National University, College of Medicine, Gwangju 501-746, Republic of Korea; ^3^Department of Orthopaedics, Gwangju KS Hospital, Gwangju, Republic of Korea; ^4^Department of Physical and Rehabilitation Medicine, Research Institute of Medical Sciences, Center for Biomedical Human Resources, Chonnam National University, Chonnam National University Medical School & Hospital, Gwangju, Republic of Korea; ^5^Department of Oral and Maxillofacial Surgery, Chonnam National University, School of Dentistry, Gwangju, Republic of Korea; ^6^Department of Orthopaedics, Mokpo Hankook Hospital, 483, Yeongsan-Ro, Mokpo City, Jeonnam, Republic of Korea

## Abstract

**Background:**

The incidence of septic arthritis of the shoulder joint is increasing as the population ages. The prevalence of shoulder infection is also increasing because of the growing use of arthroscopy and expansion of procedures in the shoulder. However, cultures do not always identify all microorganisms, even in symptomatic patients. The incidence of negative cultures ranges from 0% to 25%. Few studies have reported clinical features and treatment outcomes of culture-negative shoulder infections. This cohort study addresses culture-negative shoulder joint infections in nonarthroplasty patients. This study aimed to compare clinical characteristics and treatment outcomes of patients with culture-negative results to those with culture-positive results. Our hypothesis was that culture-negative infections would have more favorable outcomes than culture-positive infections.

**Methods:**

We retrospectively reviewed data of 36 patients (17 culture-negative and 19 culture-positive) with shoulder infections between June 2004 and March 2015. The minimum follow-up duration was 1.2 years (mean, 5 ± 3.8 years; range, 1.2-11 years). We assessed preoperative demographic data and characteristics, laboratory markers, imaging and functional scores, intraoperative findings, and postoperative findings of both groups.

**Results:**

Culture-negative patients (17/36, 47.2%) had a significantly lower occurrence of repeated surgical debridement (culture-negative vs. culture-positive: 1.2 ± 0.4 vs. 2.4 ± 1.7,* p* = 0.002) without osteomyelitis. In the multiple logistic regression analysis, the presence of osteomyelitis [odds ratio (OR) = 9.7, 95% confidence interval (CI): 1.0-91.8,* p*=0.04)] and the number of surgical debridements (OR = 5.3, 95% CI: 1.3-21.6,* p*=0.02) were significantly associated with culture-positive infections.

**Conclusions:**

Culture-negative infections without osteomyelitis are less severe than culture-positive infections. Culture-negative infections can be controlled more easily and are not necessarily a negative prognostic factor for shoulder joint infections.

## 1. Introduction

The prevalence of shoulder infections has increased recently due to the frequent use of arthroscopy and the aging population [1]. Currently, primary shoulder joint infections account for 10%-15% of all joint infections [2]. Although septic arthritis of the shoulder is rare in young and immunocompetent people, it is frequently found in the elderly [3]. Most patients who develop infections have chronic, systemic, and immunocompromising conditions, such as diabetes mellitus, blood dyscrasia, renal failure, malignancy, malnutrition, and rheumatic arthritis with a long history of corticosteroid use [1, 4–6]. The prognosis for septic arthritis of the shoulder joint is highly dependent on prompt diagnosis, cause of infection, and patients' immune system. Septic arthritis can lead to irreversible bone destruction and joint dysfunction and is occasionally a life-threatening condition, particularly in debilitated patients, making accurate diagnosis critical [7–9]. Although differential diagnosis is broad, the most serious potential cause of septic arthritis is bacterial infection [10]. Withholding antibiotic administration before culture is important to identify the causative organism from joint fluid aspirates and tissue biopsies. Despite extensive and adequate clinical, radiographic, and surgical suspicion for joint infection, the incidence of negative culture results ranges from 0% to 25%, and management with tailored antibiotics is difficult [11–16]. This cohort study aimed to assess the clinical characteristics and treatment outcomes of patients who contracted culture-negative infections after nonarthroplasty shoulder surgery. Our hypothesis was that culture-negative infections would have more favorable outcomes.

## 2. Materials and Methods

All patients provided written informed consent prior to the initiation of this study. From June 2004 to March 2015, we retrospectively reviewed data from 36 patients (18 males and 18 females) with an average age of 63.3 ± 10.2 years (range, 38-82 years) with suspected shoulder infections. Patients were divided into two groups (culture-negative, n = 17; culture-positive, n = 19) depending on culture results at the initial surgery. The minimum postoperative follow-up duration was 1.2 years (mean, 5 ± 3.8 years; range, 1.2-11 years).

The inclusion criterion was presenting in at least three out of the following classic joint infection symptoms: pain, redness, swelling, heat, and impaired range of motion. After nonarthroplasty shoulder surgery, magnetic resonance imaging (MRI) scans were performed for all patients to exclude potential structural causes of their symptoms. Synovial biopsies were harvested using punch forceps inserted through an arthroscopic cannula from representative areas of the shoulder (rotator interval, anterior capsule, and posterior capsule) to ensure equal geographic distribution. Three samples were placed in each sterile specimen container, for a total of nine specimens (3 × 3 samples per container), with removal of any foreign bodies from previous surgeries. The specimens were transported immediately at room temperature to the microbiology department, and routine culture was carried out under aseptic conditions inside a class II laminar flow biological safety cabinet to prevent aerosol contamination.

All specimens were inoculated on blood agar, MacConkey agar, and chocolate agar (Synergy Innovation, Seongnam, Korea) and were incubated in 5% CO_2_ at 35°C for 48 hours. Brucella agar, phenylethanol agar (ASAN Pharmaceutical, Hwaseong, Korea), and thioglycolate broth (Becton, Dickinson and Company, Sparks, MD, USA) were used for anaerobic cultures. The thioglycolate broth cultures were examined for turbidity daily for 14 days after inoculation. Culture plates were examined at 24 and 48 hours. The identification of microbial isolates was performed using the Vitek 2 phenotypic identification system (bioMerieux, Durham, NC, USA) and Microscan (Dade Behring, West Sacramento, CA, USA) from 2004 to 2013, and matrix-assisted laser desorption ionization-time of flight mass spectrometry (Bruker, Billerica, MA, USA) from 2014 to 2015.

**Figure 1:**
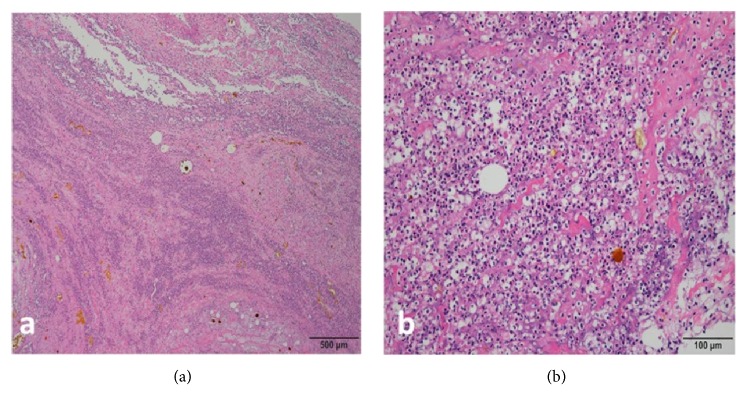
Histopathologic staining indicating acute inflammation of shoulder joint tissue in a positive culture patient. Representative images of (a) a low-power field and (b) a high-power field with more than five polymorphonuclear cells.

We evaluated demographic data, patient characteristics, preoperative standard radiographs, antibiotic administration, functional shoulder scores, arthroscopic evaluations for articular cartilage destruction, bone destruction, rotator cuff tendon degeneration, foreign suture material removal, previously used anchors, and postoperative functional scores, including the American Shoulder and Elbow Surgeons (ASES) and constant shoulder scores [10]. Patients were considered to have an infection when one of the following criteria was met: (1) microorganism growth from two separate joint tissue biopsies or joint fluid specimens, (2) presence of a communicating sinus tract with the joint, (3) histopathologic evidence of acute inflammation consistent with infection (Figure 1), or (4) when four of the following six criteria were reported: erythrocyte sedimentation rate (ESR) ≥30 mm/h, C-reactive protein (CRP) ≥10 mg/L), synovial leucocyte (WBC) count ≥2000/*μ*L, synovial neutrophil percentage (PMN%) ≥65%, presence of purulent fluid in the affected joint, microorganism isolation from a single culture of tissue or fluid, or histopathologic examination showing more than five neutrophils per high-power field [17–21].

Quantification of biomarkers in the blood (CRP, uric acid) and in the synovial fluid (lactate, uric acid) was performed to exclude gouty arthritis, which may resemble septic arthritis clinically. Synovial lactate levels above 10 mmol/L are strongly suggestive of septic arthritis, while lactate levels lower than 4.3mmol/L make it very unlikely [22, 23]. Rheumatoid factor was also measured to rule out rheumatoid arthritis.

Cultures were considered positive if organisms grew on solid media within two weeks. Growth in liquid media only was not considered consistent with infection. Joint infections were considered culture-negative if cultures obtained intraoperatively failed to grow within two weeks.

**Figure 2:**
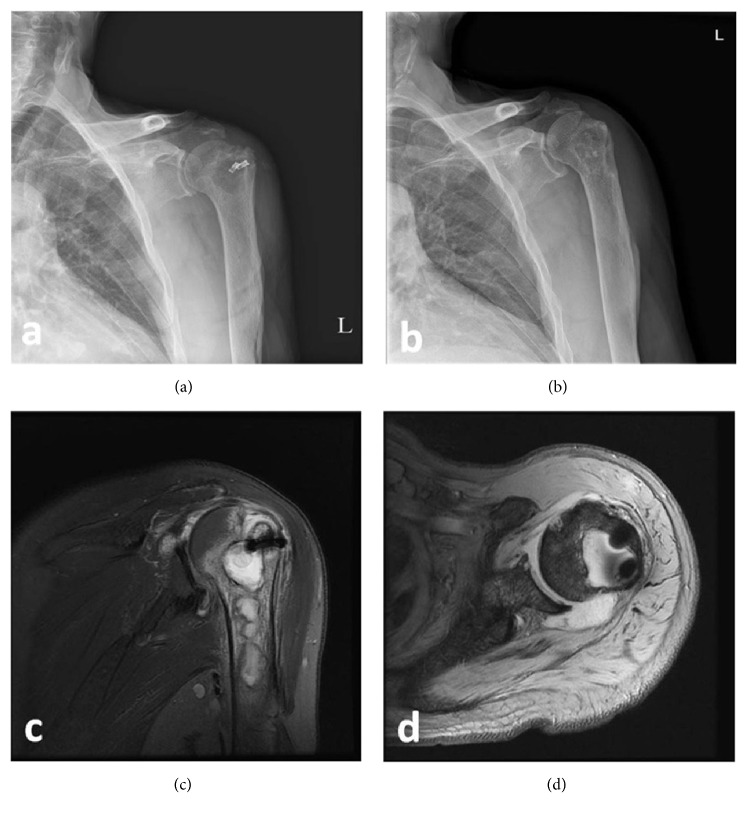
Radiographic evaluation of a 70-year-old man with a positive culture. (a) Preoperative standard X-ray (anterior-posterior) view revealing metallic anchors with an osteolytic lesion on the greater tuberosity, (b) postoperative healed bone lesion, (c) MRI-T2-coronal view showing characteristics of osteomyelitis with a multilobulated bone marrow lesion, edematous change, and synovial thickening, and (d) MRI-T2-axial view showing fluid collection in the humeral head, diffuse synovial thickening, and two metallic anchors at the greater tuberosity.

**Figure 3:**
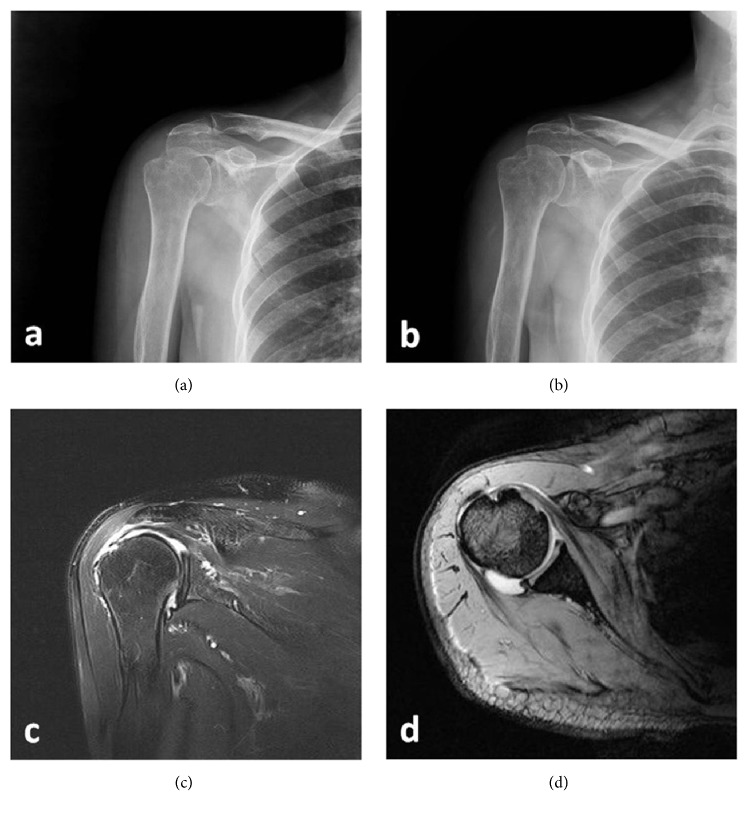
Radiographic evaluation of a 70-year-old woman with a negative culture result. (a) Preoperative standard X-ray (anterior-posterior view) showing no bony destruction of the greater tuberosity, (b) Postoperative normal proximal humerus, (c) MRI-T2-coronal view showing mild synovial thickening and joint effusion, and (d) MRI-T2-axial view showing joint fluid collection and mild synovial thickening.

Patients were also screened for osteomyelitis, a serious disease with a variety of clinically and microbiologically distinct subsets, characterized by an infection of the bone and bone marrow. We identified osteomyelitis based on the following diagnostic criteria: typical radiological findings (abnormality of the bone marrow, deep soft-tissue swelling, and/or periosteal reaction, and/or bony destruction) using standard X-rays or MRI, and pus in the bone and/or joint space [24] (Figures 2 and 3).

**Figure 4:**
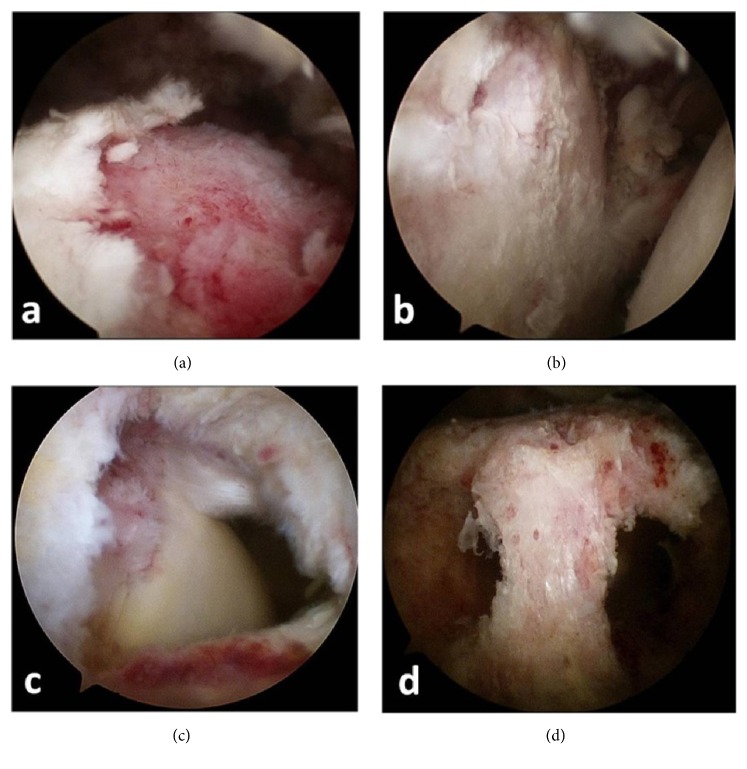
Arthroscopic evaluation of a 70-year-old man with a positive culture. (a) Arthroscopic view demonstrating destruction of the articular cartilage on the proximal humerus, (b) arthritic changes of the glenohumeral joint cartilage, (c) tendon destruction, and (d) metaphyseal bone loss from removal of previous anchors.

Prescribed antibiotics were suspended for all patients when shoulder joint infection was suspected, and arthroscopic debridement with synovectomy was performed within one week (Figure 4). Postoperative broad spectrum antibiotics were given empirically, according to the recommendation of a microbiologist. Adults were given first-generation cephalosporin (2 g cefazolin by IV every 8 hours) until culture results were available. Vancomycin (15mg/kg by IV twice daily) was used as an alternative therapy for patients allergic to cephalosporins. Repeated arthroscopic debridement was used when uncontrolled infection (e.g., persistent fever, painful effusion, laboratory signs of systemic inflammation, or positive drainage fluids) was evident, followed by concomitant antibiotic administration for at least six weeks. Fever was defined as a single oral temperature >37.8°C, repeated oral temperatures >37.2°C, or an increase in temperature of >1.1°C above the baseline temperature [25]. Treatment courses were documented for each patient.

Criteria for infection improvement were lack of pain, swelling, and wound drainage; normal serology (ESR <20 mm/h, CRP level <0.5 mg/dL); synovial leukocyte differential counts of <65% neutrophils or a leukocyte count<1.7 × 10^3^/Ul; and fewer radiographic characteristics of osteomyelitis [26].

### 2.1. Statistical Analysis

All statistical analyses were performed using SPSS Version 21.0 (SPSS Inc/IBM, Chicago, IL, USA). Chi-square and Fisher's exact tests were used to determine differences in proportions for each variable. The Shapiro Wilk test was used to check for normal distribution of data. The Independent Samples t-test was used to compare the means of continuous variables between the two groups. The Mann–Whitney U test was used for continuous variables that did not satisfy parametric assumptions. The Wilcoxon signed rank test was used for related groups of quantitative variables that were not normally distributed. Multivariate logistic regression analyses were performed to identify predictors of culture-negative joint infections. Two-tailed *p* values <0.05 were considered statistically significant.

## 3. Results

In the culture-negative group, there were nine males and eight females, with a mean age of 63.7 years (range, 50-77 years). In the culture-positive group, there were nine males and 10 females, with a mean age of 63 years (range, 38-82 years). All infections were successfully cured, regardless of culture status. Methicillin-resistant* Staphylococcus aureus* (MRSA) (9/19, 47.3%) was the most common cause of culture-positive infections, followed by methicillin-susceptible* S. aureus* (MSSA) (5/19, 26.3%), Group B* Streptococcus* (2/19, 10.5%),* Pseudomonas aeruginosa *(2/19, 10.5%), and* Candida pelliculosa* (1/19, 5.2%) (Table 1). Eleven patients had a history of ultrasound-guided injection at the shoulder joint, eight of whom were diagnosed with culture-positive infections, and three with culture-negative infections. Rotator cuff tears were found, but the exact cause was unclear. They may have been preexisting or a tenolysis effect of the infection.

There was no significant difference in age, gender, host conditions, initial diagnosis, preoperative physical symptoms, previous antibiotic treatment, synovial lactate concentration, or other laboratory data within the patients studied (Table 2).

There was a significant difference between pre- and postoperative ASES and constant shoulder scores in the culture-negative group (P=0.04), indicating that the culture-negative group showed significantly improved shoulder function postoperatively. There was no significant difference between the culture-negative and culture-positive groups, or between pre- and postoperative ASES and constant shoulder scores in the culture-positive group (Table 3).

**Figure 5:**
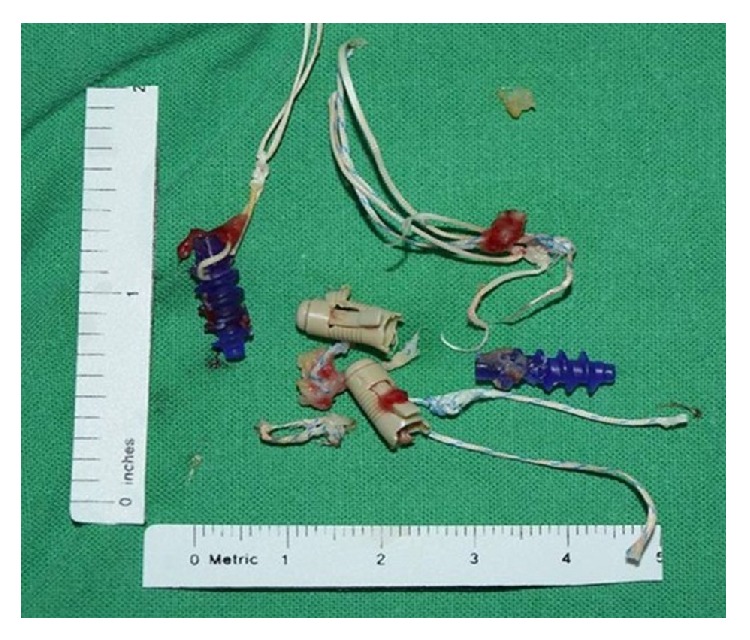
Failed anchors previously used for rotator cuff repair in some cases.

In terms of intraoperative findings, no osteomyelitis was observed in the culture-negative group. No significant difference in the presence or absence of rotator cuff tears or foreign bodies, such as anchors (Figure 5) used in previous operations, was found between the two groups. Arthroscopic debridement was effective in 29 patients. Open surgery was performed in five cases due to persistent infection.

In terms of treatment results, the culture-negative group showed significantly lower number of repeated surgical debridements compared to the culture-positive group (culture-negative vs. culture-positive, 1.2 ± 0.4 vs. 2.4 ± 1.7;* p*=0.003). Multiple logistic regression analysis revealed that the presence of osteomyelitis [odds ratio (OR) = 9.7, 95% confidence interval (CI): 1.0-91.8;* p*=0.04] and the number of surgical debridements (OR = 5.3, 95% CI: 1.3-21.6;* p*=0.02) were significant factors associated with culture-positive infections (Table 4).

## 4. Discussion

Although determining the causes of joint infection is the standard procedure for diagnosis, lack of growth in routine aerobic and anaerobic cultures is frequently encountered. Negative culture results have been reported in many joint infection series, but the clinical characteristics of such infections have not been established. Therefore, this study compared clinical characteristics and treatment results of shoulder joint infections with positive and negative culture results.

In the current study, the incidence of negative culture results was 47.2% (17/36). This was relatively high compared to incidence rates reported in other joints [11, 14]. Such differences might be due to the fact that the clinical microbiology laboratory only archives tissue samples for a short time, although this time does allow clinicians to request fungal and mycobacterial cultures if aerobic and anaerobic cultures fail to determine a pathogen [27].

All cultures in this study were monitored for 14 days to allow sufficient time for the majority of infectious organisms to grow. However, low-grade infections, such as* Propionibacterium acnes,* that are present more frequently in the shoulder joint may need prolonged incubation times of more than two weeks to yield positive results [28–33].

It is possible that the culture-negative results in this study resulted from biofilm-producing microorganisms. It is known that these microorganisms are difficult to grow under standard culture conditions [27, 34]. Recent studies reported that the demographics and outcomes of culture-negative and culture-positive patients were similar, leading to the presumption that these infections were caused by similar microorganisms [24, 25].

In the present study, there was no significant difference in age between culture-negative and culture-positive patients. Interestingly, Khan et al. found a reduced risk of infection with increasing age [35].

There was also no significant difference in sex, host conditions, infection cause, clinical symptoms, laboratory findings, or illness duration, between the two groups in this study.

Both groups had a similar history of previous antibiotic use, which was consistent with previous reports [11, 36]. It has been reported that prior antimicrobial use can reduce the sensitivity of tissue cultures [34].

The most intriguing finding of this study was that the culture-negative group showed a significantly lower need for surgical debridement, as well as a lower frequency of osteomyelitis compared to the culture-positive group. After medical and surgical management, conducted under the assumption that the culture-negative infection was due to typical bacterial pathogens, patients with culture-negative infections were more easily cured than those with culture-positive infections. These results suggest that culture-negative infections may not invade the neighboring bone, making them easier to cure.

Choi et al. also reported favorable treatment results for culture-negative patients [37]. They suggested that high vancomycin use contributed to the favorable outcome of culture-negative infections. We found that patients treated with first-generation cephalosporin had better outcomes than those treated with broad spectrum antimicrobial agents. This discrepancy indicates that the optimum therapy for culture-negative joint infections remains unknown. However, this study provides important information for patients and physicians when they encounter culture-negative results.

This study had several limitations. Due to its retrospective design and limited patient numbers, we were unable to analyze data stratified by infecting organisms. It should be noted that empirical antibiotic regimens should be evaluated based on which microorganisms are frequently causing infections. Nonetheless, the present study suggested that there was no significant difference in clinical characteristics between culture-negative and culture-positive groups. Another limitation was the culture period, which was insufficient for isolation of* Propionibacterium acnes *and other slow-growing organisms; we recommend increased incubation times of more than two weeks to isolate slow-growing organisms. Following the recommendations of our microbiologist, we did not use any local antibiotics, which could have impacted the progression of the infection and subsequent treatment.

## 5. Conclusions

Culture-negative shoulder joint infections are not necessarily a negative prognostic factor. They can be controlled more easily than culture-positive infections. Further prospective studies are required to gain additional insights into clinical characteristics and treatment outcomes of patients with culture-negative results.

## Figures and Tables

**Table 1:** Microorganisms in culture-positive shoulder infections.

Microorganism	Number of Cases (Percent)
MRSA	9 (47.3%)
MSSA	5 (26.3%)
Group B *Streptococcus*	2 (10.5%)
*Pseudomonas aeruginosa*	2 (10.5%)
*Candida pelliculosa*	1 (5.2%)
Total	19 (100%)

MRSA: Methicillin-resistant *Staphylococcus aureus*; MSSA: Methicillin-susceptible *Staphylococcus aureus.*

**Table 2:** Comparison of demographics and variables between culture-positive and culture-negative patients.

Variable	Culture Negative	Culture Positive	*P* value
	(n=17)	(n=19)	
Age, years	63.7 ± 8.6	63.0 ± 11.6	0.28
Gender			0.49
Male	9 (53%)	9 (47%)	
Female	8 (47%)	10 (53%)	
Host conditions			0.59
Uncompromised	13 (76%)	15 (79%)	
Compromised	4 (24%)	4 (21%)	
Cause			0.47
Surgery	8	9	
Injection	3	8	
Unknown	6	2	
Fever			0.53
Yes	0 (0%)	1 (5%)	
No	17 (100%)	18 (95%)	
Swelling			0.49
Yes	9 (53%)	9 (47%)	
No	8 (47%)	10 (53%)	
Evidence of heat			0.24
Yes	11 (65%)	9 (47%)	
No	6 (35%)	10 (53%)	
Draining sinus			0.21
Yes	1 (6%)	4 (21%)	
No	16 (94%)	15 (79%)	
Rotator cuff destruction			0.59
Yes	12 (71%)	13 (68%)	
No	5 (29%)	6 (32%)	
Osteomyelitis			0.01
Yes	0 (0%)	7 (32%)	
No	17 (100%)	12 (68%)	
Preoperative antibiotic treatment			0.2
Yes	4 (24%)	8 (42%)	
No	13 (76%)	11 (58%)	
Anchor			0.44
Yes	4 (24%)	6 (32%)	
No	13 (76%)	13 (68%)	
Laboratory data			
WBC (/cc)	11782 ± 3908	14952 ± 4531	0.83
ESR (mm/h)	82.5 ± 31.5	76.1 ± 28.9	0.75
CRP (mg/L)	7.8 ± 10.2	9.6 ± 9.1	0.38
Synovial lactate (mmol/L)	11.9 ± 1.1	13.8 ± 3.3	0.09
Illness duration (days)	70.7 ± 107	95.8 ± 94.5	0.14
Number of surgical debridements	1.2 ± 0.4	2.4 ± 1.7	0.003

CRP: C-reactive protein; ESR: erythrocyte sedimentation rate; WBC: white blood cell. Values are represented as (mean ± SD).

**Table 3:** Comparison of pre- and postoperative functional scores between culture-positive and culture-negative patients.

Variable	Culture Negative	Culture Positive	*P *value
	(n=17)	(n=19)	
Preoperative ASES	55.7 ± 14.7	50.8 ± 13.5	0.89
Postoperative ASES	76.0 ± 19.1	77.5 ± 11.2	0.85

*P* value	0.04	0.18	

Preoperative constant score	53.6 ± 25.1	56.0 ± 33.9	0.69
Postoperative constant score	75.8 ± 19.8	80.5 ± 17.7	0.69

*P* value	0.04	0.18	

ASES: American shoulder and elbow surgeons score; values are represented as (mean ± SD).

**Table 4:** Influential factors of culture-negative infections, based on multiple logistic regression analysis.

Variables	Odds Ratio	*P* value	Confidence Interval	
			Lower limit	Upper limit
Osteomyelitis	9.7	0.04	1.0	91.8
Number of surgical debridements	5.3	0.02	1.3	21.6

## Data Availability

Datasets used and/or analyzed during the current study are available from the corresponding author upon reasonable request.
